# Supplementary material for: SARS-CoV-2 infection and vaccination elicit distinct pharyngeal mucosal B cell responses in children
Source: Nat Commun. 2026 May 22;17:6924. doi: 10.1038/s41467-026-72996-3 (PMC13388998; doi:10.1038/s41467-026-72996-3)
Supplement: Supplementary file 1 — Supplementary Information [file 41467_2026_72996_MOESM1_ESM.pdf]

SARS-CoV-2 infection and vaccination elicit distinct pharyngeal mucosal B cell responses in children

Qin Xu[1], Lihong Shi[1], Center for Human Immunology[*], Can Liu[2], Juraj Kabat[3], Hengameh Behzadpour[4], Lela Kardava[5], Tovah E. Markowitz[6], Margery Smelkinson[3], Kenneth B. Hoehn[7], Clarisa M. Buckner[5], Dominic P. Golec[1], Lorenza Bellusci[8], Gabrielle Grubbs[8], Sara Pourhashemi[8], Juanjie Tang[8], Asya Khleborodova[6], Martha Kirby[9], Rachel Sparks[10], Andrew J. Martins[2], John S. Tsang[2,11], Susan Moir[6], Surender Khurana[8], Pamela Mudd[4,12], Pamela L. Schwartzberg[1#], Kalpana Manthiram[1#]

[1] Cell Signaling and Immunity Section, Laboratory of Immune System Biology, National Institute of Allergy and Infectious Diseases, National Institutes of Health, Bethesda, Maryland, United States of America
[2] Department of Immunobiology; Yale Center for Systems and Engineering Immunology, Yale School of Medicine, New Haven, Connecticut, United States of America
[3] Biological Imaging Section, Research Technologies Branch, National Institute of Allergy and Infectious Diseases, National Institutes of Health, Bethesda, Maryland
[4] Division of Pediatric Otolaryngology, Children's National Hospital, Washington, District of Columbia, United States of America
[5] B-cell Immunology Section, Laboratory of Immunoregulation, National Institute of Allergy and Infectious Diseases, National Institutes of Health, Bethesda, Maryland, United States of America
[6] Integrated Data Sciences Section, Research Technologies Branch, National Institute of Allergy and Infectious Diseases, National Institutes of Health, Bethesda, Maryland, United States of America
[7] Department of Biomedical Data Science and Dartmouth Cancer Center, Geisel School of Medicine at Dartmouth, Hanover, New Hampshire, United States of America
[8] Division of Viral Products, Center for Biologics Evaluation and Research, Food and Drug Administration, Silver Spring, Maryland, United States of America
[9] National Human Genome Research Institute, National Institutes of Health, Bethesda, Maryland, United States of America
[10] Laboratory of Immune System Biology, National Institute of Allergy and Infectious Diseases, National Institutes of Health, Bethesda, Maryland, United States of America
[11] Department of Biomedical Engineering, Yale University, New Haven, Connecticut, United States of America
[12] Division of Otolaryngology, Department of Surgery, George Washington University School of Medicine and Health Sciences, Washington, District of Columbia, United States of America

* A list of authors and their affiliations appears at the end of the manuscript.
# Correspondence: kalpana.manthiram@nih.gov (K.M.); pamela.schwartzberg@nih.gov (P.L.S.)

Supplementary Figures:

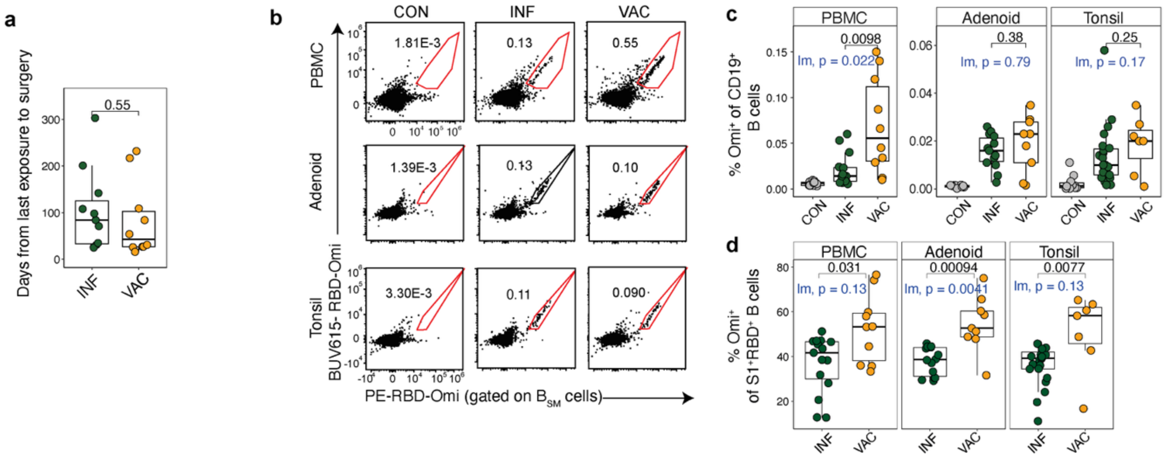

Supplementary Figure 1. SARS-CoV-2-specific B cells in the pharyngeal tissues and blood post-vaccination and post-infection

(a) Days from the latest immunologic exposure (vaccination or infection) to surgery. Comparisons of the 2 groups were done with the two-sided Mann-Whitney U test. N denotes the number of individual subjects per group: INF N = 11, VAC N = 10.

(b) Representative flow plots of omicron-specific (Omi$^+$) B cells in PBMCs, adenoids and tonsils of CON, INF and VAC participants.

(c) Percentages of Omi$^+$ CD19$^+$ B cells in PBMCs, adenoids and tonsils of CON, INF and VAC participants (same samples as Figure 1f).

(d) Percentages of Omi$^+$ cells among WA-1 S1$^+$RBD$^+$ B cells in PBMCs, adenoids and tonsils (same samples as in Figure 1f).

P values obtained from linear model correcting for participant ages (in blue) or from two-sided Mann-Whitney U test (in black) are shown in panels c and d. *P* < 0.05 were considered significant. Box plots (a, c and d) show the median (center line) and interquartile range (25th–75th percentiles; box bounds) with whiskers extending to the most extreme values within 1.5× the interquartile range. Individual data points are shown.

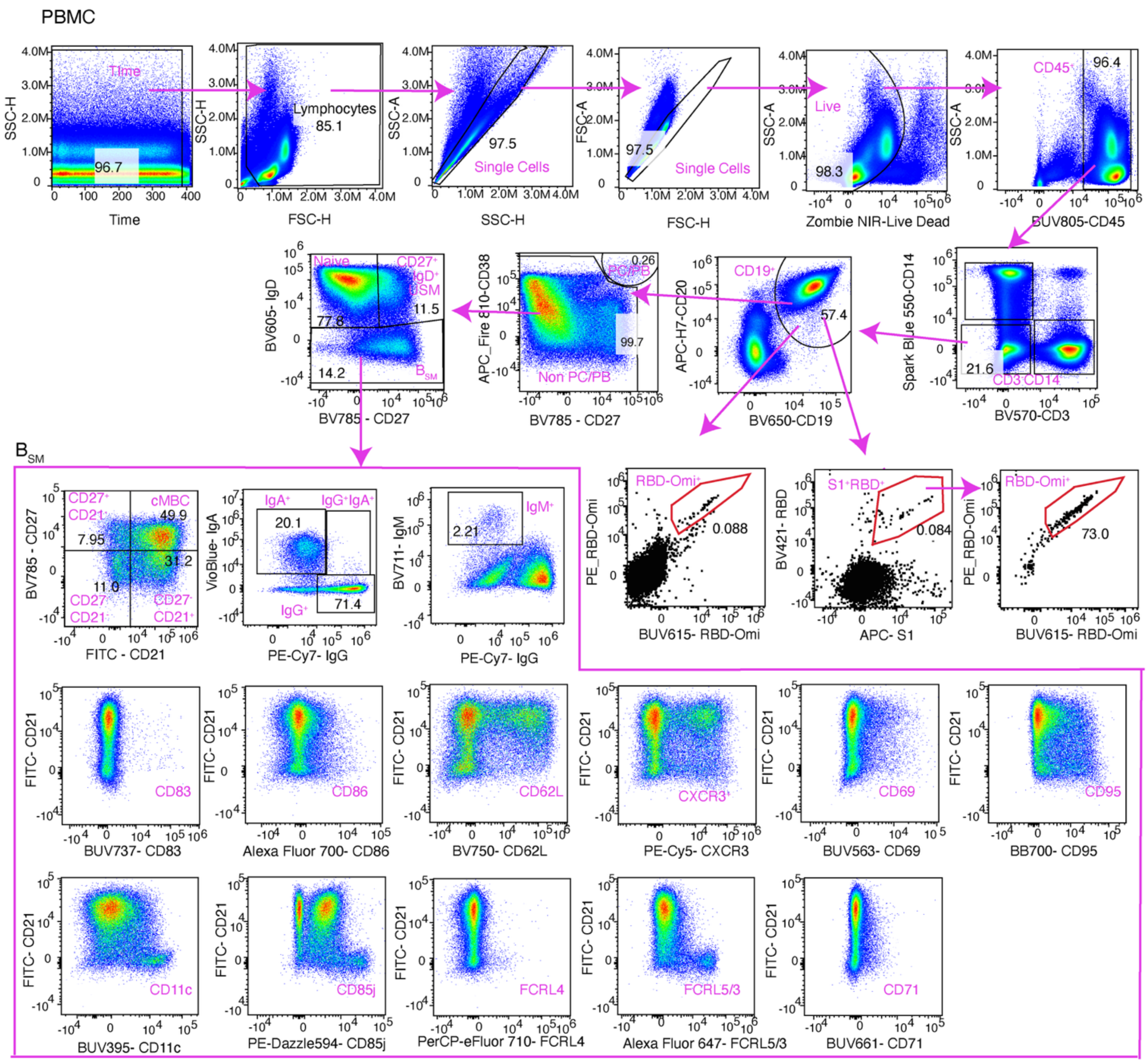

Supplementary Figure 2. Gating strategy for SARS-CoV-2-specific B cells in PBMCs

Gating strategy for identifying and characterizing SARS-CoV-2 antigen-specific B cells ($S1^{+}RBD^{+}$ or $RBD\text{-}Omi^{+}$) in peripheral blood mononuclear cells (PBMC).
PC = plasma cells; PB = plasmablast; USM = unswitched memory B cells; B_{SM} = switched memory B cells; cMBC = conventional memory B cells

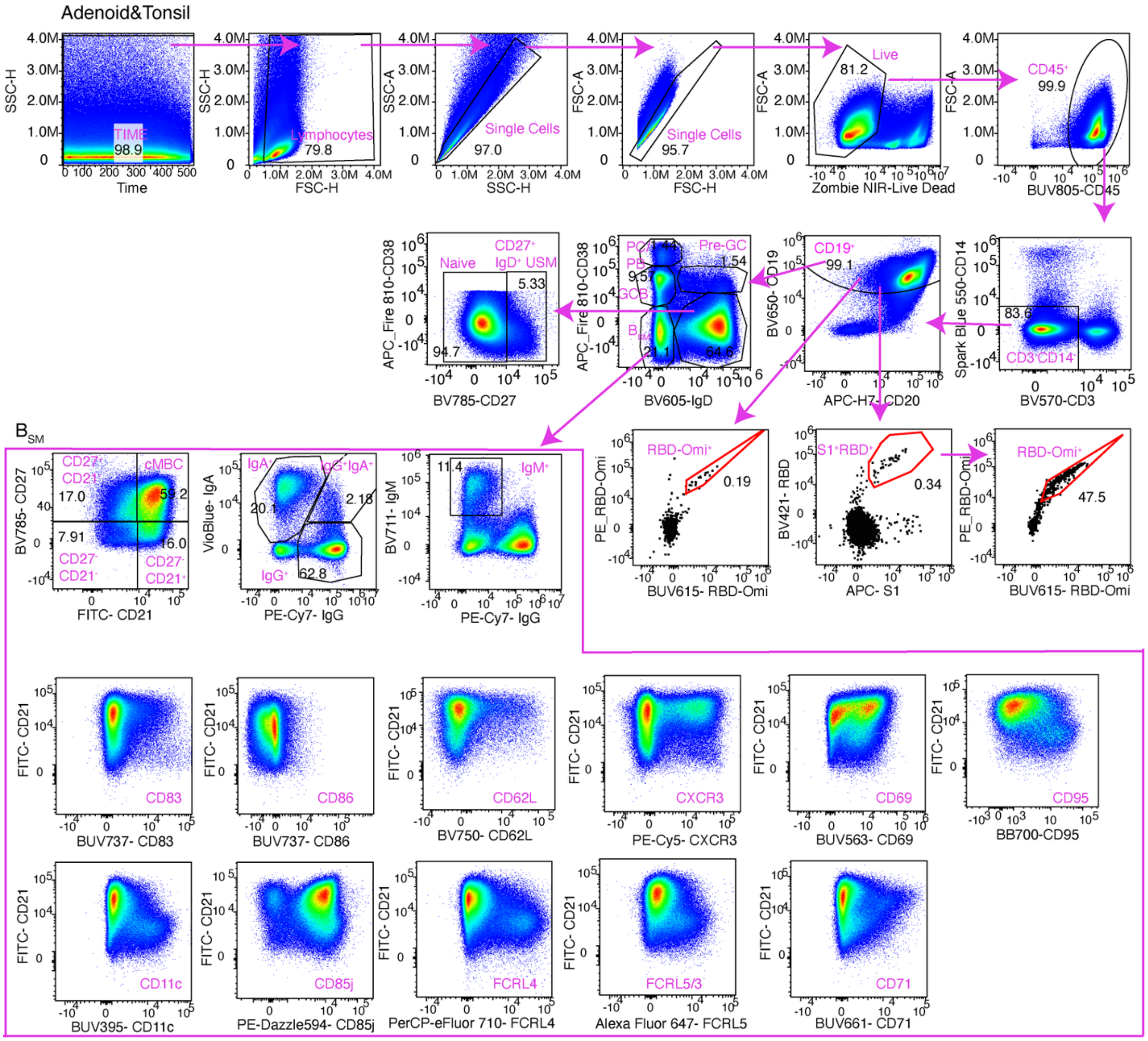

Supplementary Figure 3. Gating strategy for SARS-CoV-2-specific B cells in adenoids and tonsils.

Gating strategy for identifying and characterizing SARS-CoV-2 antigen-specific ($S1^+RBD^+$ and RBD-Omi^+) B cells in adenoids and tonsils.

PC = plasma cell; PB = plasmablast; GCB = germinal center B cells; B_{SM} = switched memory B cells; USM = unswitched memory B cells; cMBC = conventional memory B cells

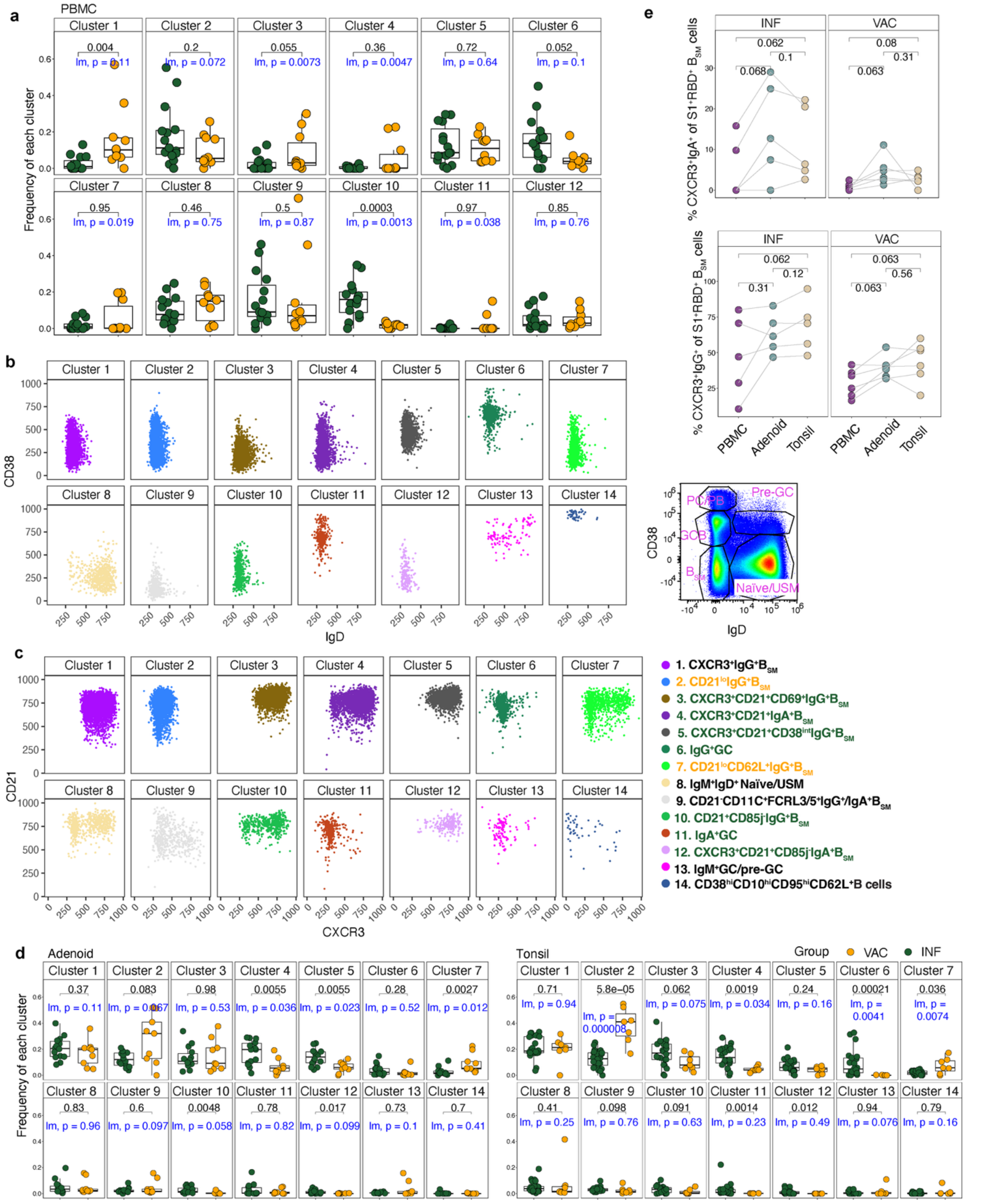

Supplementary Figure 4. Unsupervised analyses of S1$^+$RBD$^+$ B cells from PBMC and tissues of INF and VAC participants

(a) Comparison of frequencies of each cluster among S1⁺RBD⁺ B cells in PBMC by group (INF N = 15, VAC N = 10, Supplemental Data 5).
(b) Expression of IgD and CD38 among clusters (shown in Figure 4a-b) from unsupervised analyses of S1⁺RBD⁺ B cells from tissues. Right: representative flow plot of CD38 and IgD indicating different B cell populations.
(c) Expression of CXCR3 and CD21 among clusters (shown in Figure 4a-b) from unsupervised analyses of S1⁺RBD⁺ B cells from tissues.
(d) Comparisons of cluster frequencies among S1⁺RBD⁺ B cells in INF and VAC groups according to tissue type. N denotes the number of individual subjects per group (adenoid: INF N = 14, VAC N = 9; tonsil: INF N = 22, VAC N = 7; Supplemental Data 6).
(e) Percentages of CXCR3⁺IgA⁺ (upper panel) and CXCR3⁺IgG⁺ (lower panel) cells among S1⁺RBD⁺ B_{SM} in subjects with matched PBMCs, adenoids and tonsils from INF and VAC groups (INF = 5, VAC = 6). *P* values were calculated with paired two-sided Wilcoxon signed ranks test.

P values (a and d) obtained from linear model correcting for participant ages (in blue) and from two-sided Mann-Whitney U test (in black) are shown. $P < 0.05$ was considered significant. Box plots (a and d) show the median (center line) and interquartile range (25th–75th percentiles; box bounds) with whiskers extending to the most extreme values within 1.5× the interquartile range. Individual data points are shown.

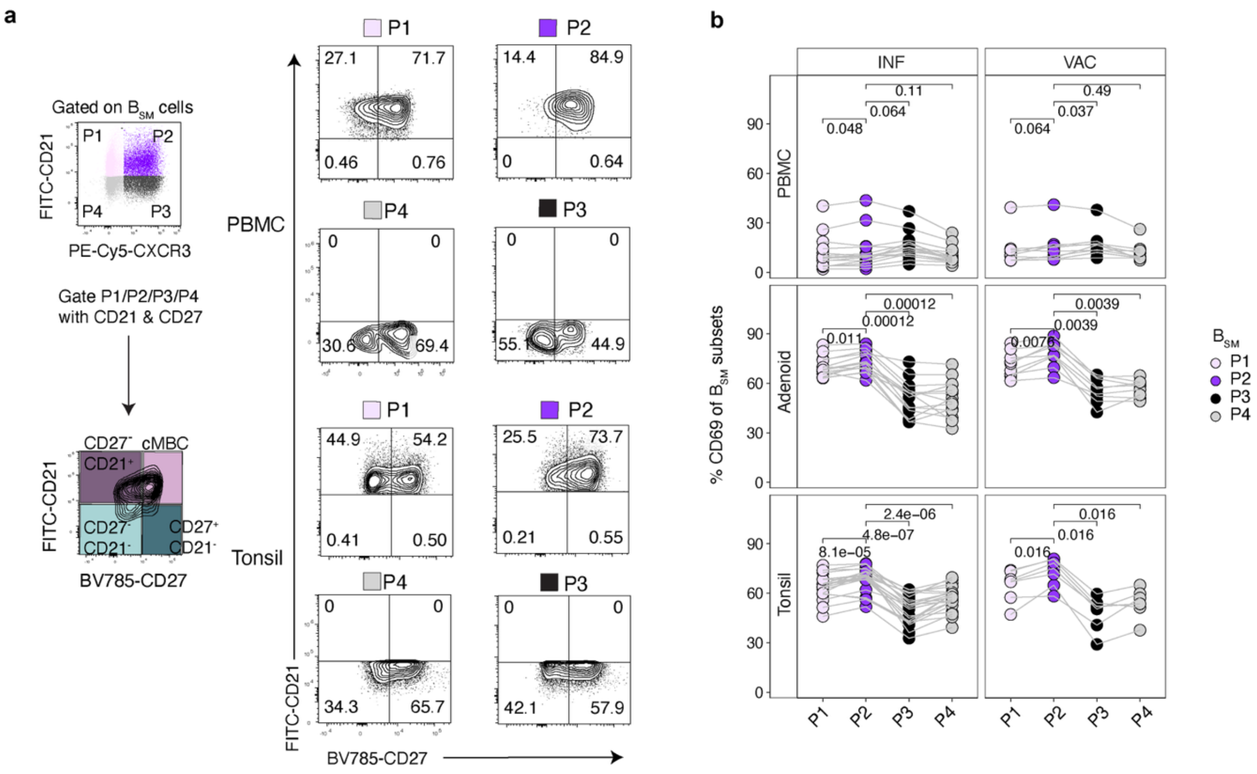

Supplementary Figure 5. Characterization of P2 ($CXCR3^+CD21^+$) B_{SM}

(a) Representative gating of P1 to P4 B_{SM} based on CD27 and CD21 expression in PBMC and tonsil to define cMBC and other MBC subsets.

(b) Percentage of $CD69^+$ cells among bulk P1 to P4 B_{SM} populations in PBMCs, adenoids and tonsils of INF and VAC participants. N denotes the number of individual subjects per group (PBMC: INF N = 15, VAC N = 10; adenoid: INF N = 14, VAC N = 9; tonsil: INF N = 22, VAC N = 7). $CD69^+$ P2 population was compared to the other types of B_{SM}. *P* values were calculated with paired two-sided Wilcoxon signed ranks test. $P < 0.05$ were considered significant. Individual data points are shown.

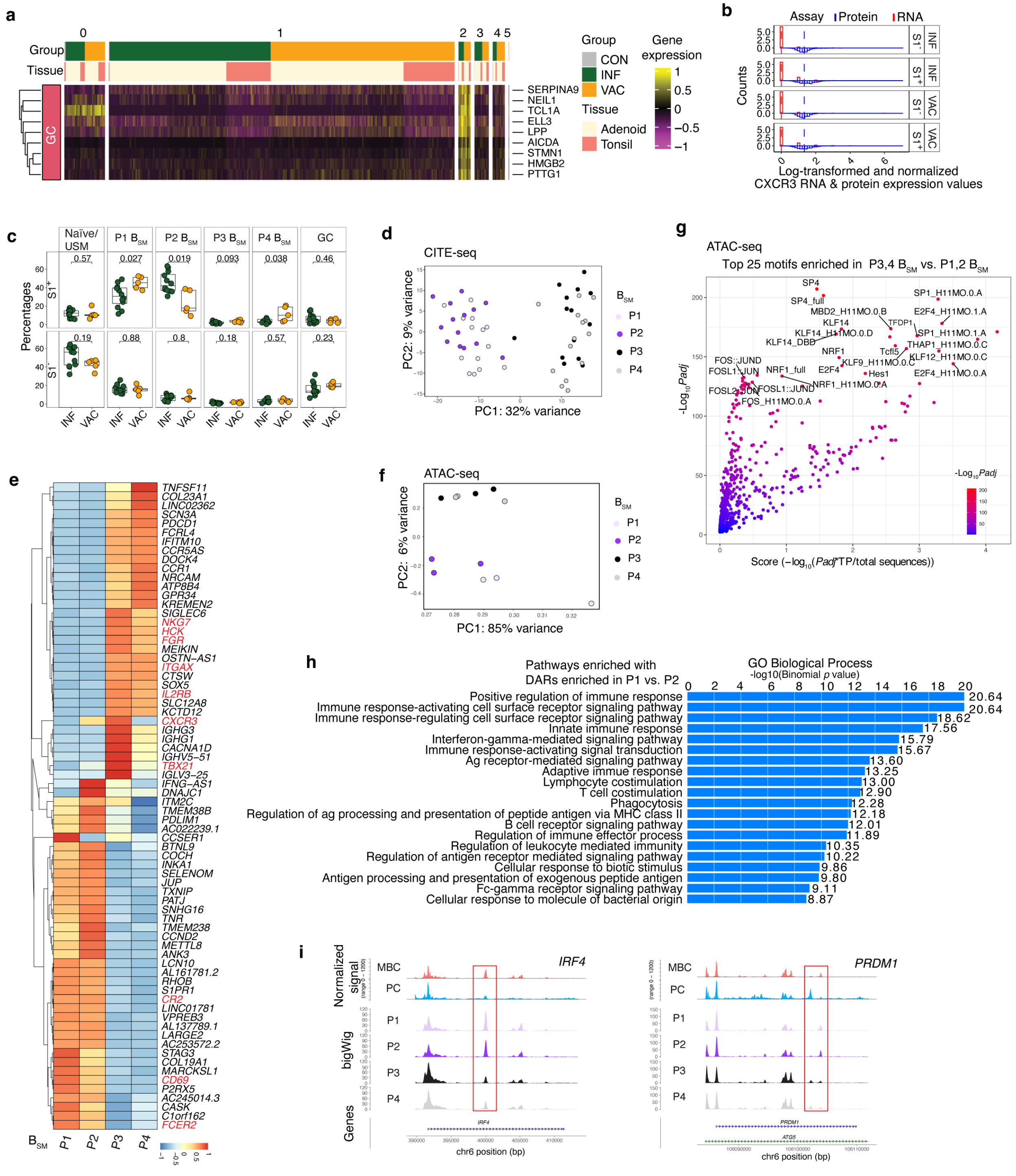

Supplementary Figure 6. Characterization of tissue S1$^+$ B cells with CITE-seq and ATAC-seq

(a) Heatmap showing expression of GCB signature genes among S1$^+$ B cells by cluster (samples are from sample set 306-4 described in Supplemental Data 8).

(b) RNA counts for *CXCR3* (red) and log-transformed and normalized CXCR3 surface protein levels (blue) from CITE-seq in $S1^+$ and $S1^-$ B cells in INF and VAC tissues.

(c) Percentages of each B cell subset manually gated based on surface antibody expression among $S1^+$ or $S1^-$ cells of INF and VAC participants, compared with two-sided Mann-Whitney U test.

(d) PCA of P1 to P4 B_{SM} from tissue based on transcriptome from CITE-seq. Each point represents an B_{SM} subset from an individual's tissue sample.

(e) Heatmap of top differentially expressed genes among P1 to P4 tissue B_{SM} subsets from CITE-seq. Panels a-d use same samples as Figure 5e.

(f) PCA of epigenomic profiles from ATAC-seq from P1 to P4 tonsil B_{SM}, showing the variation in library-size normalized counts of consensus peaks (≥2 samples). Each point represents a B_{SM} subset from an individual tonsil sample (N = 3 tonsils, listed in Supplemental Data 2).

(g) Top 25 enriched motifs based on differential peaks identified by comparing peaks combined from P3 and P4 populations with those from P1 and P2. True positives (TP) refer to accurately identified motif occurrences within the given sequence dataset.

(h) Pathway enrichment analysis by Genomic Regions Enrichment of Annotations Tool (GREAT) using DARs enriched in P1 compared to P2.

(i) ATAC-seq genome tracks at the *IRF4* and *PRDM1* loci in sorted tonsil P1 to P4 B_{SM} subsets and memory B cells (MBC) and plasma cells (PC) from published tonsil sc-ATAC-seq dataset[90].

Box plots (c) show the median (center line) and interquartile range (25th–75th percentiles; box bounds) with whiskers extending to the most extreme values within 1.5× the interquartile range. Individual data points are shown. $P < 0.05$ were considered significant. Individual data points are shown.

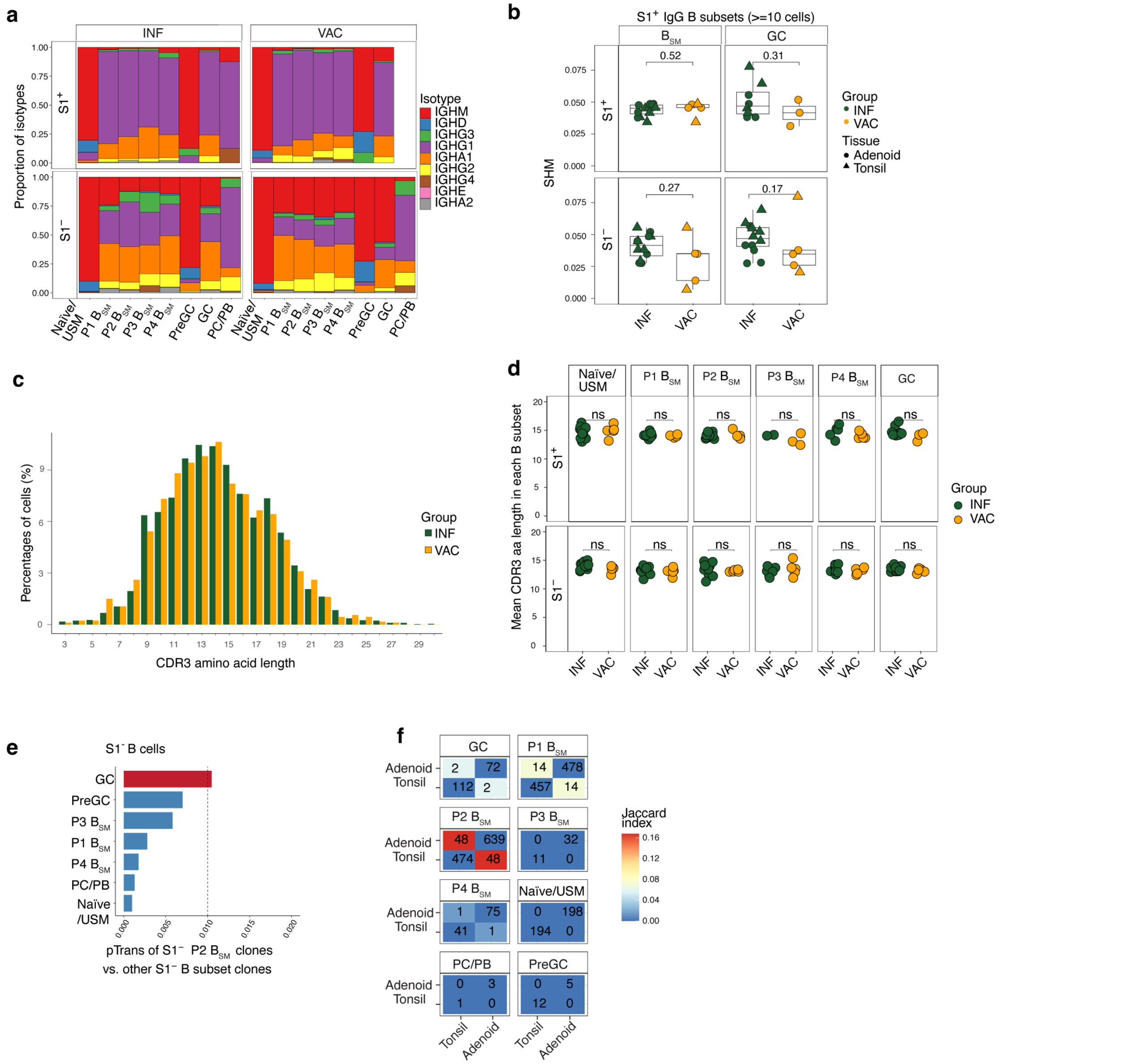

Supplementary Figure 7. Single cell BCR repertoire analysis of S1^{+} and S1^{-} B cells in INF and VAC tissues

(a) Composition of isotypes in each B cell subset among S1^{+} or S1^{-} cells from PBMCs, adenoids, and tonsils of VAC and INF participants. Mean percentages are shown (same samples as Figure 6a).

(b) Median SHM frequency among tissue specific S1^{+}IgG^{+} B$_{SM}$ or S1^{+}IgG^{+} GCB populations of VAC versus INF participants (adenoid and tonsil are both shown). Only samples with at least 10 cells in analyzed population are included. N denotes the number of individual subjects per group (B$_{SM}$: S1^{+} INF N = 12, VAC N = 5; S^{-} INF N = 12, VAC N = 5; GC: S1^{+} INF N = 8, VAC = 3, S1^{-} INF N = 12, VAC N = 5).

(c) CDR3 amino acid lengths of $S1^+$ cells of VAC and INF adenoids and tonsils (same samples as Figure 6a).

(d) Mean CDR3 amino acid lengths of $S1^+$ cells from B cell subsets of VAC versus INF adenoids and tonsils (same samples as Figure 6a).

(e) Degree of clonal overlap measured with pairwise transition index (pTrans) between clones in $S1^-$ P2 B_{SM} and clones in other $S1^-$ B cell subsets in tonsils and adenoids (same samples as Figure 6a).

(f) Heatmap of clonal overlap between adenoid and tonsil from donors with both tissues included (N = 5). Off-diagonal elements are colored by the Jaccard index of clonal overlap between the two tissues and are labeled by the raw number of overlapping clones. Diagonal elements are labeled by the total number of clones within a particular subset.

P values were calculated with two-sided Wilcoxon signed ranks test (paired) in b and d. Box plots (b and d) show the median (center line) and interquartile range (25th–75th percentiles; box bounds) with whiskers extending to the most extreme values within 1.5× the interquartile range. Individual data points are shown. $P < 0.05$ were considered significant.

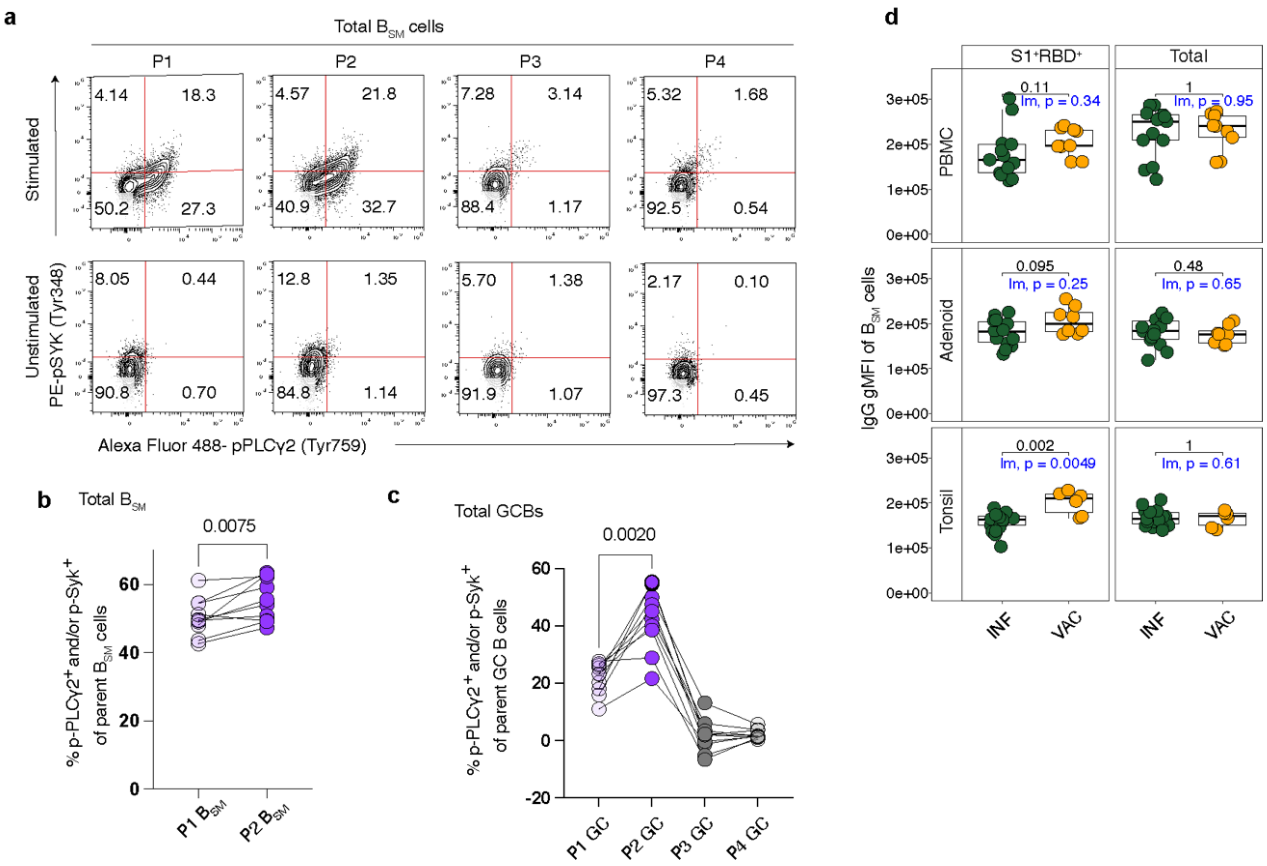

Supplementary Figure 8. Response to BCR stimulation among B_{SM} subsets

(a) Phosphorylation of Syk and PLCγ2 in bulk (total) B_{SM} subsets with and without 2-minute stimulation with soluble anti-IgG, -IgA, and -IgM. Representative plots are shown (N = 10 tonsils, listed in Supplemental Data 2).

(b-c) Percentage of cells expressing either or both p-Syk and/or p-PLCγ2 among P1 versus P2 total B_{SM} (b) or P1 to P4 subpopulations in total GCB (c) after BCR stimulation (percentages of p-Syk and/or p-PLCγ2 from untreated conditions were subtracted from stimulated conditions) (N = 10 tonsils).

(d) gMFI of surface IgG on $S1^+RBD^+$ and bulk B_{SM} of PBMCs, adenoids and tonsils from INF and VAC participants. Samples with ≥ 10 IgG^+ $S1^+RBD^+$ B_{SM} cells were used in this analysis (PBMC: INF N = 13, VAC N = 9; adenoid: INF N = 14, VAC N = 8; tonsil: INF N = 21, VAC N = 6).

P values were calculated with two-sided Wilcoxon signed ranks test (paired) in b and c. In panel d, *p* values obtained from linear model correcting for participant ages (in blue) and from two-sided Mann-Whitney U test (in black) are shown. Box plots (d) show the median (center line), interquartile range (25th–75th percentiles; box bounds), with whiskers extending to the most extreme values within 1.5× the interquartile range. Individual data points are shown. $P < 0.05$ were considered significant.

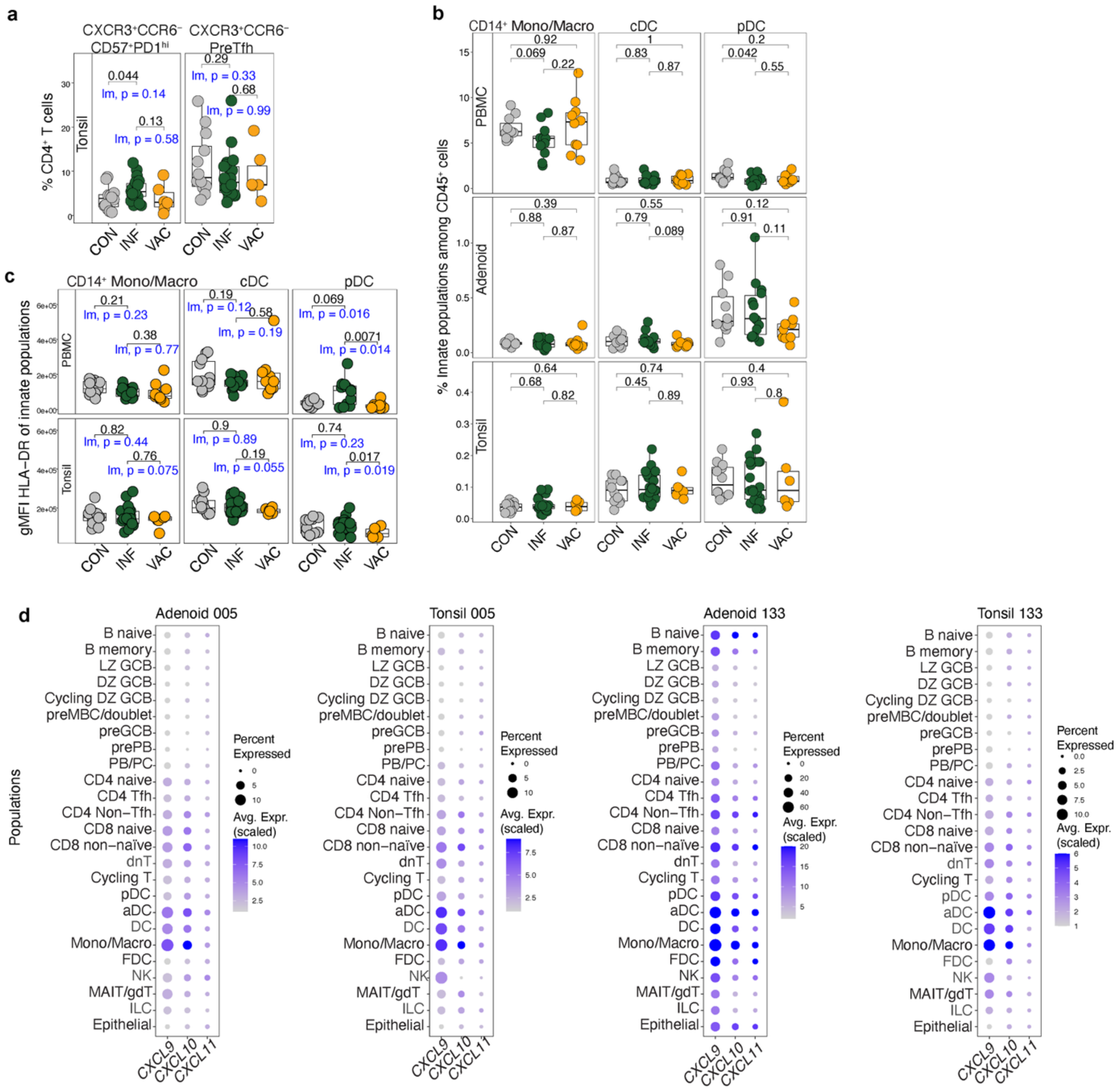

Supplementary Figure 9. Spatial analyses of CXCR3^{+} B$_{SM}$ in mucosal pharyngeal tissues

(a) Percentages of CXCR3^{+}CCR6^{-}CD57^{+}PD1^{hi} and CXCR3^{+}CCR6^{-} pre-Tfh (gated on CD25^{-}CD4^{+} cells as shown in Supplemental Figure 10) among CD4^{+} T cells in CON, INF and VAC tonsils. N denotes the number of individual subjects per group (CON N = 12, INF N = 22, VAC N = 6, listed in Supplemental Data 2).

(b) Percentages of CD14^{+} monocytes/macrophages, cDC and pDC among CD45^{+} cells in CON, INF and VAC PBMCs, adenoids and tonsils. N denotes the number of individual subjects per group (PBMC CON N = 11, INF N = 12, VAC N = 10; adenoid CON N = 10, INF N = 14, VAC N = 9; tonsil CON N = 12, INF N = 22, VAC N = 6).

(c) gMFI of HLA-DR expression on $CD14^+$ monocytes/macrophages, cDCs, and pDCs among CON, INF, and VAC PBMCs and tonsils (same PBMC and tonsil samples as in panel b).
(d) Scaled expression of *CXCL9*, *CXCL10*, and *CXCL11* in each cell type from the spatially sequenced samples (N = 4 tissues).

In panels a and c, *p* values obtained from linear model correcting for participant ages (in blue) and from two-sided Mann-Whitney U test (in black) are shown. In b, *p* values obtained from two-sided Mann-Whitney U test are shown. Box plots (a-c) show the median (center line) and interquartile range (25th–75th percentiles; box bounds) with whiskers extending to the most extreme values within 1.5× the interquartile range. Individual data points are shown. Individual data points are shown. $P < 0.05$ were considered significant.

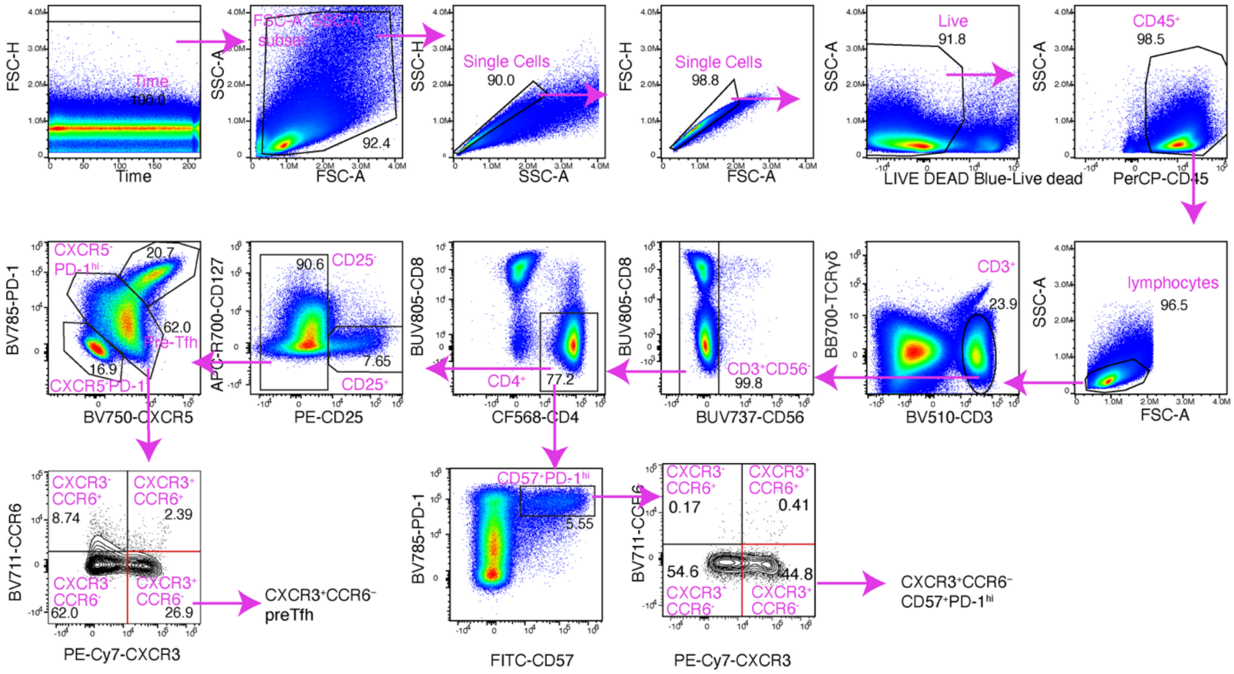

Supplementary Figure 10. Gating strategy for CD4$^+$ T cells in tissues

Gating strategy for CD4$^+$ T cells in adenoid and tonsil.
GC-Tfh = germinal center T follicular helper cell; pre-Tfh = pre-T follicular helper cell

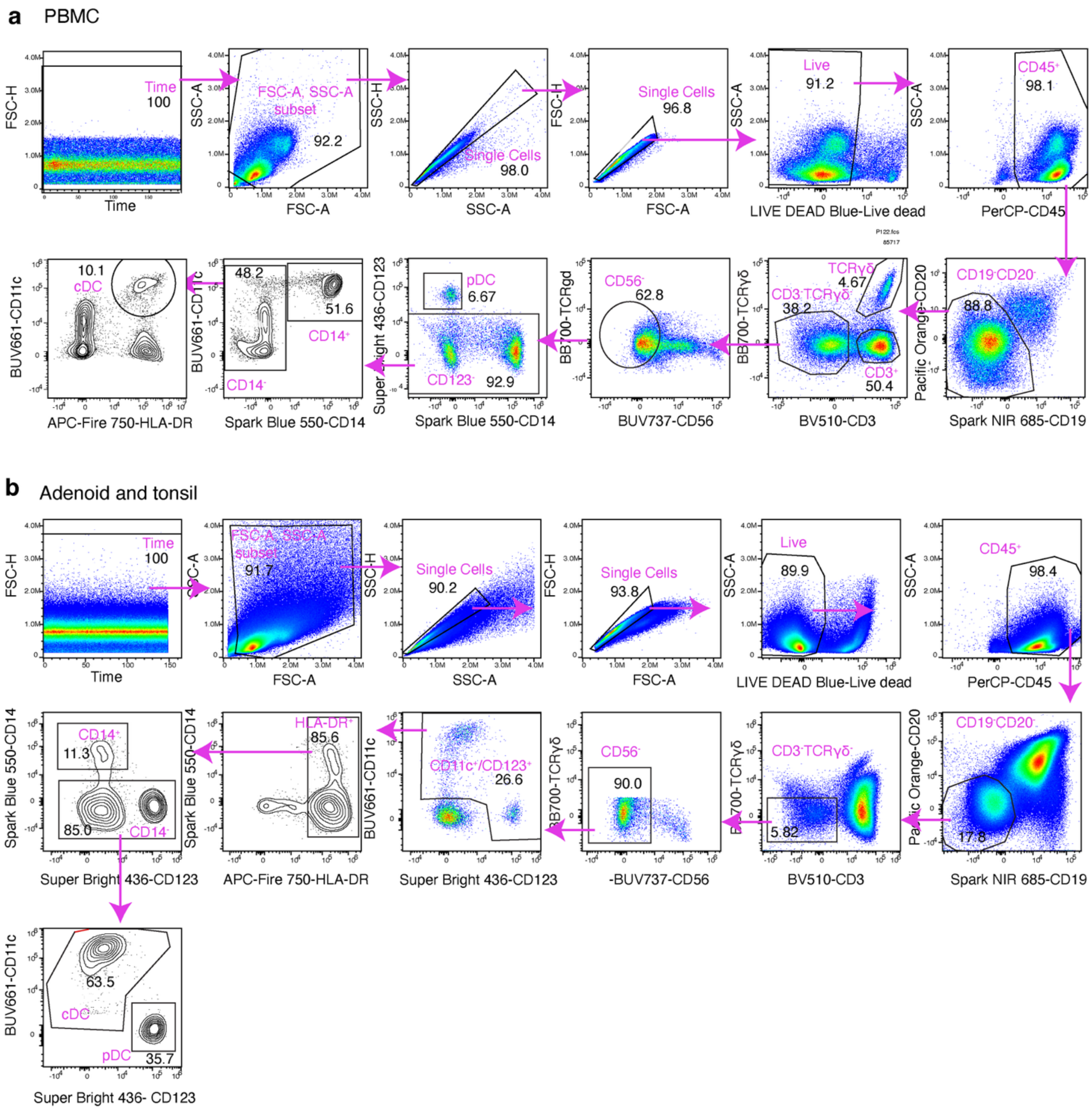

Supplementary Figure 11. Gating strategy for innate immune cell populations

(a) Gating strategy for innate populations in PBMC.

(b) Gating strategy for innate populations in adenoid and tonsil. cDC = conventional dendritic cells; pDC = plasmacytoid dendritic cells

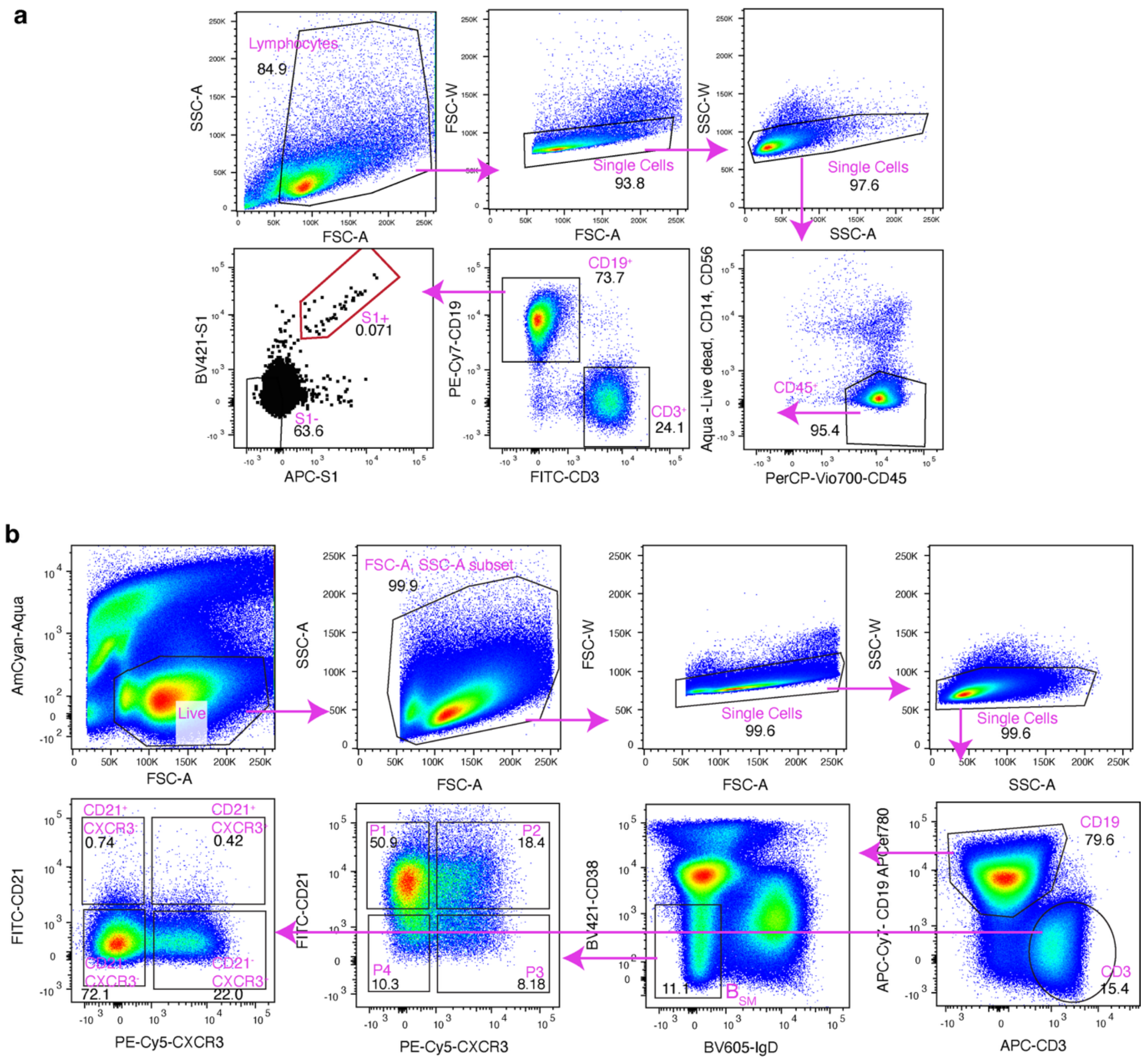

Supplementary Figure 12. B cell sorting strategies

(a) S1^{+} and S1^{-} B cell sorting strategy for CITE-seq assay.

(b) P1-P4 B$_{SM}$ sorting strategy for ATAC-seq assay and *in vitro* plasma blast cell differentiation and proliferation assay. Gating of CD21 and CXCR3 among CD3^{+} T cells was used to position the gates for B$_{SM}$. B$_{SM}$ = switched memory B cells

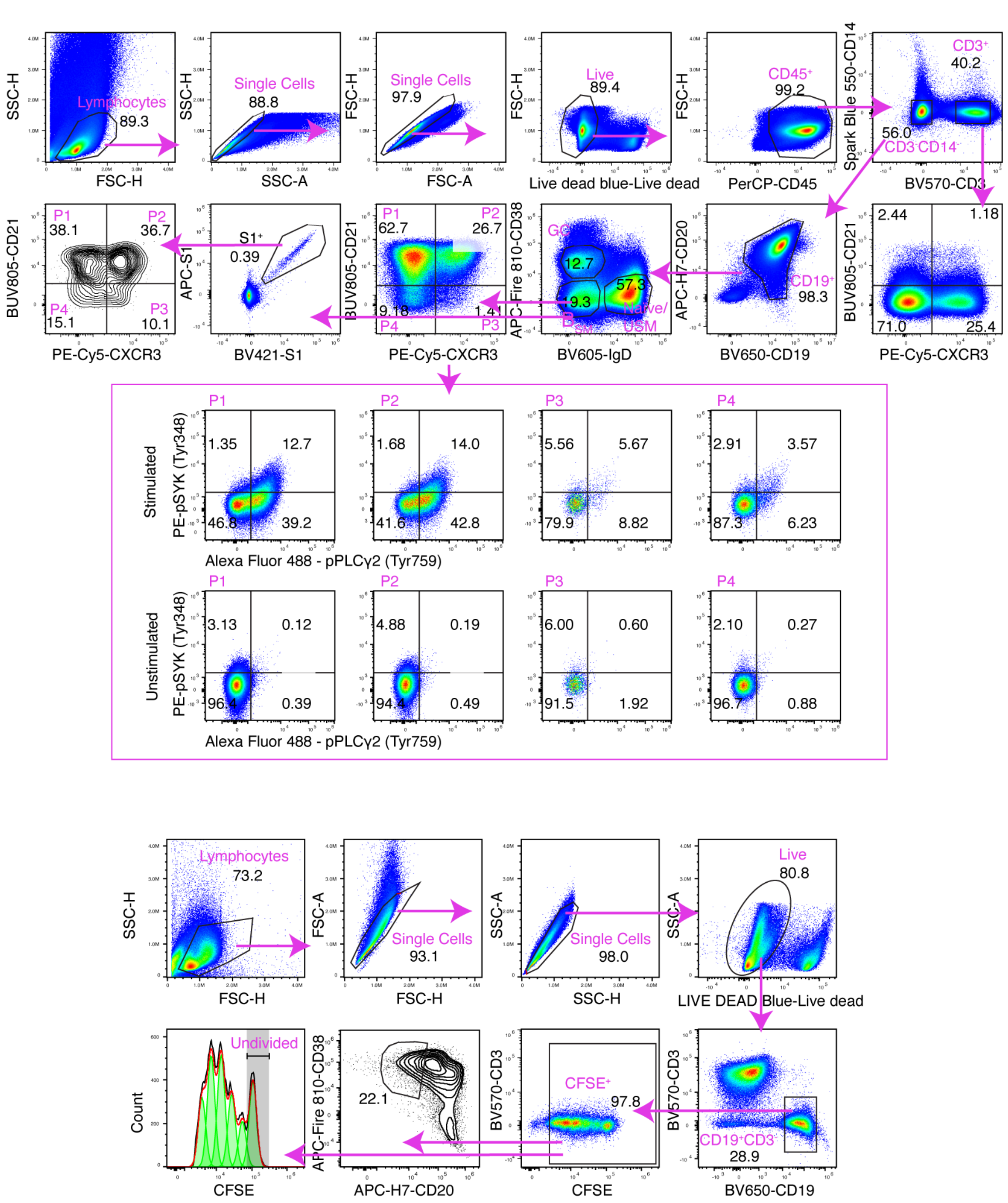

Supplementary Figure 13. Gating strategy for B cell functional assays

(a) Gating strategy for B cell phosphorylation assay with and without stimulation with anti-human IgA/G/M.

(b) Gating strategy for *in vitro* plasma blast cell differentiation and proliferation assay. CFSE = carboxyfluorescein succinimidyl ester; B_{SM} = switched memory B cells; GC = germinal center

Supplementary Note 1. README

Our study assesses immune responses to SARS-CoV-2 in the tonsils, adenoids, and peripheral blood of children who have received a COVID-19 mRNA vaccine and who were previously infected with SARS-CoV-2. We used high dimensional flow cytometry and CITE-seq to characterize SARS-CoV-2-specific B cells as well as ATAC-seq to characterize B cell subpopulations. We analyzed the flow cytometry data with unsupervised clustering and performed statistical analyses with a linear model to compare the immune profiles of subjects who were vaccinated and infected. For CITE-seq, we assessed surface markers, transcriptome and BCR repertoire of SARS-CoV-2-specific B cells and B cell subpopulations of interest.

Software dependencies and operating systems (including version numbers):

Operating systems: macOS Apple M3Max, Sonoma 14.8.2

Core software: R version 4.4.2 (2024-10-31)

IDE: Rstudio (2023.12.1.402)

R platform dependencies
    Platform: aarch64-apple-darwin20
    Running under: macOS Sonoma 14.8.2
Matrix products: default
BLAS:
/System/Library/Frameworks/Accelerate.framework/Versions/A/Frameworks/vecLib.framework/Versions/A/libBLAS.dylib
LAPACK: /Library/Frameworks/R.framework/Versions/4.4-arm64/Resources/lib/libRlapack.dylib; LAPACK version 3.12.0

locale:
[1] en_US.UTF-8/en_US.UTF-8/en_US.UTF-8/C/en_US.UTF-8/en_US.UTF-8

time zone: America/New_York
tzcode source: internal

attached base packages:
```
[1] stats     graphics  grDevices utils     datasets  methods   base
```

other attached packages:
```
[1] dowser_2.3        Startrac_0.1.0     SeuratObject_5.1.0 sp_2.2-0          alakazam_1.3.0     ggplot2_3.5.2
```

loaded via a namespace (and not attached):
```
 [1] RColorBrewer_1.1-3        rstudioapi_0.17.1         jsonlite_2.0.0            shape_1.4.6.1             magrittr_2.0.3
 [6] ggbeeswarm_0.7.2          farver_2.1.2              GlobalOptions_0.1.2       fs_1.6.6                  fields_16.3
[11] zlibbioc_1.52.0           vctrs_0.6.5               Rsamtools_2.22.0          DelayedMatrixStats_1.28.0 ggtree_3.14.0
[16] rstatix_0.7.2             airr_1.5.0                S4Arrays_1.6.0            progress_1.2.3            dynamicTreeCut_1.63-1
[21] broom_1.0.9               SparseArray_1.6.0         Formula_1.2-5             gridGraphics_0.5-1        pracma_2.4.4
[26] parallelly_1.43.0         KernSmooth_2.23-24        plyr_1.8.9                impute_1.80.0             GenomicAlignments_1.42.0
[31] igraph_2.1.4              lifecycle_1.0.4           iterators_1.0.14          pkgconfig_2.0.3           Matrix_1.7-1
[36] R6_2.6.1                  GenomeInfoDbData_1.2.13   MatrixGenerics_1.18.0     future_1.40.0             clue_0.3-66
[41] digest_0.6.37             aplot_0.2.8               colorspace_2.1-1          patchwork_1.3.0           S4Vectors_0.44.0
[46] GenomicRanges_1.58.0      ggpubr_0.6.1              progressr_0.15.1          phylotate_1.3             httr_1.4.7
[51] abind_1.4-8               compiler_4.4.2            withr_3.0.2               doParallel_1.0.17         backports_1.5.0
```

```
 [56] BiocParallel_1.40.2        carData_3.0-5              viridis_0.6.5              dendextend_1.19.0          maps_3.4.2.1
 [61] ggsignif_0.6.4             MASS_7.3-61                DelayedArray_0.32.0        rjson_0.2.23               tools_4.4.2
 [66] vipor_0.4.7                beeswarm_0.4.0             ape_5.8-1                  future.apply_1.11.3        quadprog_1.5-8
 [71] glue_1.8.0                 nlme_3.1-166               grid_4.4.2                 gridBase_0.4-7             reshape2_1.4.4
 [76] cluster_2.1.6              ade4_1.7-23                generics_0.1.4             seqinr_4.2-36              gtable_0.3.6
 [81] tzdb_0.5.0                 tidyr_1.3.1                shazam_1.2.0               data.table_1.16.2          hms_1.1.3
 [86] car_3.1-3                  XVector_0.46.0             BiocGenerics_0.52.0        markdown_1.13              stringr_1.5.1
 [91] ggrepel_0.9.6              foreach_1.5.2              pillar_1.11.0              limma_3.62.1               yulab.utils_0.2.0
 [96] spam_2.11-1                circlize_0.4.16            dplyr_1.1.4                treeio_1.30.0              lattice_0.22-6
[101] ks_1.14.3                  tidyselect_1.2.1           ComplexHeatmap_2.22.0      SingleCellExperiment_1.28.1 Biostrings_2.74.1
[106] gridExtra_2.3              IRanges_2.40.0             SummarizedExperiment_1.36.0 scattermore_1.2            RhpcBLASctl_0.23-42
[111] stats4_4.4.2               Biobase_2.66.0             diptest_0.77-1             statmod_1.5.0              sscVis_0.1.0
[116] matrixStats_1.5.0          stringi_1.8.7              UCSC.utils_1.2.0           lazyeval_0.2.2             ggfun_0.1.8
[121] yaml_2.3.10                codetools_0.2-20           tibble_3.3.0               ggplotify_0.1.2            cli_3.6.5
[126] moduleColor_1.8-4          dichromat_2.0-0.1          Rcpp_1.1.0                 GenomeInfoDb_1.42.1        globals_0.17.0
[131] png_0.1-8                  ggrastr_1.0.2              parallel_4.4.2             readr_2.1.5                prettyunits_1.2.0
[136] mclust_6.1.1               dotCall64_1.2              sparseMatrixStats_1.18.0   bitops_1.0-9               phangorn_2.12.1
[141] listenv_0.9.1              mvtnorm_1.3-2              viridisLite_0.4.2          tidytree_0.4.6             scales_1.4.0
[146] purrr_1.1.0                crayon_1.5.3               GetoptLong_1.0.5           rlang_1.1.6                fastmatch_1.1-6
[151] cowplot_1.2.0
```
