# Supplementary material for: SARS-CoV-2 infection and vaccination elicit distinct pharyngeal mucosal B cell responses in children
Source: Nat Commun. 2026 May 22;17:6924. doi: 10.1038/s41467-026-72996-3 (PMC13388998; doi:10.1038/s41467-026-72996-3)
Supplement: Supplementary file 2 — Description of Additional Supplementary Files [file 41467_2026_72996_MOESM2_ESM.pdf]

Description of Additional Supplementary Files

Supplementary Data 1. Characteristics of each group

Supplementary Data 2. Participant characteristics and summary of samples used in immune profiling

Supplementary Data 3. Serologic test summary and percentages of $S1^+RBD^+$ B cells

Supplementary Data 4. Class switched memory B cell (B_{SM}) characteristics

Supplementary Data 5. Frequency of each cluster (PBMC)

Supplementary Data 6. Frequency of each cluster (tonsil and adenoid)

Supplementary Data 7. Germinal center B cells

Supplementary Data 8. Samples sequenced with CITE-seq

Supplementary Data 9. Top 40 differential genes by comparing $S1^+$ P1 and P2 B_{SM} ($S1^+$ P2 B_{SM} vs. $S1^+$ P1 B_{SM})

Supplementary Data 10. Top 20 differential peaks by comparing P1 and P2 B_{SM} (P2 B_{SM} vs. P1 B_{SM})

Supplementary Data 11. Reagent Tables
