# Supplementary material for: SARS-CoV-2 infection and vaccination elicit distinct pharyngeal mucosal B cell responses in children
Source: Nat Commun. 2026 May 22;17:6924. doi: 10.1038/s41467-026-72996-3 (PMC13388998; doi:10.1038/s41467-026-72996-3)
Supplement: Supplementary file 5 — Reporting Summary [file 41467_2026_72996_MOESM5_ESM.pdf]

nature portfolio

Corresponding author(s): Kalpana Manthiram, Pamela Schwartzberg

last updated by author(s): 3-20-2026

Reporting Summary

Nature Portfolio wishes to improve the reproducibility of the work that we publish. This form provides structure for consistency and transparency in reporting. For further information on Nature Portfolio policies, see our Editorial Policies and the Editorial Policy Checklist.

Statistics

For all statistical analyses, confirm that the following items are present in the figure legend, table legend, main text, or Methods section.

n/a	Confirmed	
☐	☒	The exact sample size (*n*) for each experimental group/condition, given as a discrete number and unit of measurement
☐	☒	A statement on whether measurements were taken from distinct samples or whether the same sample was measured repeatedly
☐	☒	The statistical test(s) used AND whether they are one- or two-sided<br>*Only common tests should be described solely by name; describe more complex techniques in the Methods section.*
☐	☒	A description of all covariates tested
☐	☒	A description of any assumptions or corrections, such as tests of normality and adjustment for multiple comparisons
☐	☒	A full description of the statistical parameters including central tendency (e.g. means) or other basic estimates (e.g. regression coefficient) AND variation (e.g. standard deviation) or associated estimates of uncertainty (e.g. confidence intervals)
☐	☒	For null hypothesis testing, the test statistic (e.g. *F*, *t*, *r*) with confidence intervals, effect sizes, degrees of freedom and *P* value noted<br>*Give P values as exact values whenever suitable.*
☒	☐	For Bayesian analysis, information on the choice of priors and Markov chain Monte Carlo settings
☒	☐	For hierarchical and complex designs, identification of the appropriate level for tests and full reporting of outcomes
☐	☒	Estimates of effect sizes (e.g. Cohen's *d*, Pearson's *r*), indicating how they were calculated

Our web collection on statistics for biologists contains articles on many of the points above.

Software and code

Policy information about availability of computer code

Data collection	Participants' clinical data were collected and managed in REDCap web database platform.<br>Flow cytometric data were collected on a Cytek Aurora with manufacturer's software (SpectroFlo V1.1).<br>Neutralizing assay was acquired from One-Glo luciferase assay system (Promega).<br>S1+/S1- B cells, P1-P4 switched memory B cells were sorted from adenoid and tonsil on FACSAria Fusion with manufacturer's software (BD FACSDiva Software V8.0.2).<br>scRNA+CITEseq+BCRseq libraries were sequenced from Illumina NovaSeq or Xplus platform.<br>ATACseq were acquired from Illumina NovaSeq platform.
Data analysis	Details of all packages and software were provided in manuscript. Scripts were deposited at https://github.com/kalpanamanthiram/COVID-19-Vaccination.

For manuscripts utilizing custom algorithms or software that are central to the research but not yet described in published literature, software must be made available to editors and reviewers. We strongly encourage code deposition in a community repository (e.g. GitHub). See the Nature Portfolio guidelines for submitting code & software for further information.

Data

Policy information about availability of data

All manuscripts must include a data availability statement. This statement should provide the following information, where applicable:

- Accession codes, unique identifiers, or web links for publicly available datasets
- A description of any restrictions on data availability
- For clinical datasets or third party data, please ensure that the statement adheres to our policy

The CITE-seq and ATAC-seq data are deposited to dbGAP under accession number phs004475.v1.p1.

Research involving human participants, their data, or biological material

Policy information about studies with human participants or human data. See also policy information about sex, gender (identity/presentation), and sexual orientation and race, ethnicity and racism.

Reporting on sex and gender	This data is contained in Supplementary Data 2.
Reporting on race, ethnicity, or other socially relevant groupings	This data is contained in Supplementary Data 2.
Population characteristics	This data is contained in Supplementary Data 2.
Recruitment	We recruited 21 children who underwent tonsillectomy and/or adenoidectomy at Children's National Hospital (CNH) in Washington, DC, USA and also received at least one dose of a COVID-19 mRNA vaccine. The first 17 participants were recruited from December 2021 to April 2022, and the remaining were recruited from August 2022 to September 2022. Because not all tissues or blood were available from each subject, we collected a total of 21 blood samples, 19 adenoids, and 18 tonsils from these 21 participants.
Ethics oversight	This study was approved by the Institutional Review Board (IRB) at Children's National Hospital (IRB protocol number 00009806). Written informed consent was obtained from parent/guardians of all enrolled participants, and assent was obtained from minor participants over 7 years of age.

Note that full information on the approval of the study protocol must also be provided in the manuscript.

Field-specific reporting

Please select the one below that is the best fit for your research. If you are not sure, read the appropriate sections before making your selection.

☒ Life sciences ☐ Behavioural & social sciences ☐ Ecological, evolutionary & environmental sciences

For a reference copy of the document with all sections, see nature.com/documents/nr-reporting-summary-flat.pdf

Life sciences study design

All studies must disclose on these points even when the disclosure is negative.

Sample size	No statistical methods were used to predetermine sample size. Small sample sizes are typical CITE-seq and spatial transcriptomic data.
Data exclusions.	Data exclusions criteria for each analysis were included in the figure legends and methods.
Replication.	CITE-seq findings were consistent with results from flow cytometry, and several were further validated using in vitro functional assays or supported by ATAC-seq, as described in the manuscript. Paired tonsil and adenoid samples from two donors were used for imaging and Xenium spatial transcriptomics. Due to the nature of the study design, replication was not feasible.
Randomization	No randomization.
Blinding	Researchers were not blinded as participants were queried about exposure history to the COVID-19-exposure status or vaccination.

Behavioural & social sciences study design

All studies must disclose on these points even when the disclosure is negative.

Study description	
Research sample	
Sampling strategy	

Data collection

Timing

Data exclusions

Non-participation

Randomization

Ecological, evolutionary & environmental sciences study design

All studies must disclose on these points even when the disclosure is negative.

Study description

Research sample

Sampling strategy

Data collection

Timing and spatial scale

Data exclusions

Reproducibility

Randomization

Blinding

Did the study involve field work? ☐ Yes ☐ No

Field work, collection and transport

Field conditions

Location

Access & import/export

Disturbance

Reporting for specific materials, systems and methods

We require information from authors about some types of materials, experimental systems and methods used in many studies. Here, indicate whether each material, system or method listed is relevant to your study. If you are not sure if a list item applies to your research, read the appropriate section before selecting a response.

Materials & experimental systems

n/a	Involved in the study	
☐	☒	Antibodies
☐	☒	Eukaryotic cell lines
☒	☐	Palaeontology and archaeology
☒	☐	Animals and other organisms
☐	☒	Clinical data
☒	☐	Dual use research of concern

Methods

n/a	Involved in the study	
☒	☐	ChIP-seq
☐	☒	Flow cytometry
☒	☐	MRI-based neuroimaging

Antibodies

Antibodies used

All antibodies, sources and research resource identifiers listed

1.29c B cell panel
Number Reagent Source Catalog number Clone Dilution (PBMC, ul/200ul) Dilution (Adenoid and Tonsil, ul/200ul)
1 Anti-CD11c-BUV395 BD 563787 B-ly6 3 ul 4.5 ul
2 Anti-CD71-BUV661 BD 749818 L01.1 0.5 ul 1.6 ul
3 Anti-CXCR3-PE-Cy5 BD 551128 1C6/CXCR3 5ul 5ul
4 Anti-CD45-BUV805 BD 612891 HI30 2 ul 3.3 ul
5 Anti-CD62L-BV750 BD 747422 DREG-56 0.6 ul 0.6 ul
6 Anti-FCRL5/FCRL3-AF647 BD 564343 307307 2.5 ul 4.5 ul
7 Anti-CD86-AF700 BD 561124 2331 (FUN-1) 1 ul 2.5 ul
8 Anti-CD20-APC-H7 BD 560734 2H7 2 ul 3.3 ul
9 Anti-CD10-BV510 BD 563032 HI10a 2 ul 1.6 ul
10 Anti-CD19-BV650 BD 563226 SJ25C1 3 ul 7.5 ul
11 Anti-CD95-BB700 BD 340480 DX2 1 ul 0.25 ul 0.25ul
12 Anti-IgG-PE-Cy7 BD 561298 G18-145 2 ul 6.5 ul
13 Anti-CD3-BV570 BioLegend 300436 UCHT1 3 ul 3.3 ul
14 Anti-IgD-BV605 BioLegend 348232 IA6-2 2 ul 2.5 ul
15 Anti-IgM-BV711 BioLegend 314540 MHM-88 2 ul 5 ul
16 Anti-CD27-BV785 BioLegend 302832 O323 3 ul 1.6 ul
17 Anti-CD14-Spark Blue 550 BioLegend 367148 63D3 2.5 ul 3.3 ul
18 Anti-CD21-FITC BioLegend 354910 Bu32 2 ul 1.6 ul
19 Live dead dye-Zombie NIR BioLegend 423106 0.25 ul 0.25 ul
20 CoV2-RBD-Biotin BioLegend 793906 2.5 ul of conjugated antibody 2.5 ul of conjugated antibody
21 SAv-BV421 BioLegend 405225 conjugated with CoV2-RBD-Biotin
22 CoV2-S1-Biotin BioLegend 793806 5 ul of conjugated antibody 5 ul of conjugated antibody
23 SAv-APC ThermoFisher S32362 conjugated with CoV2-S1-Biotin
24 Anti-CD38-APC-Fire810 BioLgend 303550 HIT2 1 ul 1 ul
25 Anti-IgA-VioBlue Miltenyi 130-113-479 IS11-8E10 2.5 ul 1.3 ul
26 Anti-CD85j-Super bright 436 ThermoFisher 62-5129-42 HP-F1 2.5 ul 6.5 ul
27 Anti-FCRL4-PerCP-eFluor710 ThermoFisher 46-3079-42 413D12 5 ul 2.4 ul
28 SAv-PE ThermoFisher S21388 conjugated with Omicron-RBD-Biotin
29 Omicron-RBD-Biotin Acro SPD-C82E4 2.5 ul of conjugated antibody 2.5 ul of conjugated antibody
30 SAv-BUV615 BD 613013 conjugated with Omicron-RBD-Biotin

2. **37c flow panel:**
Number Reagent Source Catalog Number Clone Dilution (PBMC, ul/180ul) Dilution (Adenoid and Tonsil, ul/180ul)
1 Anti-CD141-BB515 BD 566017 1A4 2.5 ul 2.5 ul
2 Anti-CD16-BUV496 BD 612944 3G8 0.6 ul 0.6 ul
3 Anti-TCR γδ-BB700 BD 745944 11F2 1 ul 4 ul
4 Anti-CD11c-BUV661 BD 612967 B-ly6 3.5 ul 3.5 ul
5 Anti-IgG-BV605 BD 563246 G18-145 5 ul 5 ul
6 Anti-CD127-APC-R700 BD 565185 HIL-7R-M21 6 ul 5 ul
7 Anti-IgD-BV480 BD 566138 IA6-2 0.6 ul 1 ul
8 Anti-CD56-BUV737 BD 612766 NCAM16.2 3.5 ul 3.5 ul
9 Anti-CXCR5-BV750 BD 747111 RF8B2 1.2 ul 1.2 ul
10 Anti-CD8-BUV805 BD 612889 SK1 1.2 ul 1.2 ul
11 Anti-CD103-PE/Dazzle-594 BioLegend 350224 Ber-ACT8 5ul 2.5ul
12 Anti-CD45RA-BUV395 BD 740315 5H9 0.3 ul 0.6 ul
13 SAv-BUV615 BD 613013 0.9 ul 0.9 ul
14 Anti-CD14-Spark Blue 550 BioLegend 367148 63D3 2.5 ul 2.5 ul
15 Anti-CD25-PE BioLegend 302606 BC96 10 ul 10 ul
16 Anti-CD28-BV650 BioLegend 302946 CD28.2 2.5 ul 5 ul

Antibodies used

17 Anti-CXCR3-PE-Cy7 BioLegend 353720 G025H7 5 ul 5 ul
18 Anti-PD-1-BV785 BioLegend 329930 EH12.2H7 5 ul 2.5 ul
19 Anti-CCR6-BV711 BioLegend 353436 G034E3 1.2 ul 1.2 ul
20 Anti-CCR7-BV421 BioLegend 353208 G043H7 5 ul 5 ul
21 Anti-CD19-Spark NIR 685 BioLegend 302270 HIB19 1.2 ul 2.5 ul
22 Anti-CD38-APC/Fire 810 BioLegend 303550 HIT2 1 ul 1 ul
23 Anti-CD57-FITC BioLegend 359604 HNK-1 1.2 ul 1.2 ul
24 Anti-CD1c-Alexa Fluor 647 BioLegend 331510 L161 5 ul 1.2 ul
25 Anti-HLA-DR-APC-Fire 750 BioLegend 307658 L243 2.5 ul 2.5 ul
26 Anti-IgM-BV570 BioLegend 314517 MHM-88 2.5 ul 4 ul
27 Anti-CD69-BUV563 BD 748764 FN50 1ul 1ul
28 Anti-CD3-BV510 BioLegend 317332 OKT3 5 ul 5 ul
29 Anti-CD21-PerCP CY5.5 BioLegend 354908 Bu32 1.2 ul 2.5 ul
30 Anti-CD4-CF568 Cytek R7-20041 SK3 1.2 ul 1.2 ul
31 Anti-IgA-Biotin Jackson ImmunoResearch 109-066-011 Polyclonal 5 ul 5 ul
32 Anti-CD123-Super Bright 436 ThermoFisher 62-1239-42 6H6 3.5 ul 3.5 ul
33 Anti-CD95-PE-Cy5 ThermoFisher 15-0959-42 DX2 0.6 ul 0.6 ul
34 Anti-CD45-PerCP ThermoFisher MHCD4531 HI30 1.2 ul 1.2 ul
35 Anti-CD20-Pacific Orange ThermoFisher MHCD2030 HI47 2.5 ul 7.5 ul
36 Anti-CD161-eFluor 450 ThermoFisher 48-1619-42 HP-3G10 5 ul 5 ul
37 Anti-CD27-APC ThermoFisher 17-0279-42 O323 5 ul 2.5 ul
38 Live dead dye-Blue ThermoFisher L23105 0.225 ul 0.225

3. Transcription Factor panel
Number Reagent Source Catalog number Clone Dilution (PBMC, ul/200ul) Dilution (Adenoid and Tonsil, ul/200ul)
1 Anti-CD11c-BUV395 BD 563787 B-ly6 3 ul 4.5 ul
2 Anti-CD69-BUV563 BD 748764FN50 1ul 1ul
3 Anti-CD71-BUV661 BD 749818 L01.1 0.5 ul 1.6 ul
4 Anti-CD10-BUV737 BD 612826 HI10a 1.6ul 1.6ul
5 Anti-BCL6-PE BD 561522 K112-91 8ul 8ul
6 Anti-CXCR5-BV750 BD 747111 RF8B2 1ul 1ul
7 Anti-FCRL5/FCRL3-AF647 BD 564343 CD307e/c 5 ul 2.4 ul
8 Anti-Ki67-AF700 BD 561277 B56 2ul 2ul
9 Anti-CD20-APC-H7 BD 560734 2H7 2 ul 3.3 ul
10 Anti-IgG-BV510 BD 563247 G18-145 2.5ul 2.5ul
11 Anti-CD19-BV650 BD 563226 SJ25C1 3 ul 7.5 ul
12 Anti-CD95-BB700 BD 566542 DX2 0.125ul 0.125ul
13 Anti-CD183(CXCR3)-PE-Cy5 BD 551128 1C6/CXCR3 5ul 5ul
14 Anti-CD3-BV570 BioLegend 300436 UCHT1 3 ul 3.3 ul
15 Anti-IgD-BV605 BioLegend 348232 IA6-2 2 ul 2.5 ul
16 Anti-IgM-BV711 BioLegend 314540 MHM-88 2 ul 5 ul
17 Anti-CD27-BV785 BioLegend 302832 O323 3 ul 1.6 ul
18 Anti-CD14-Spark Blue 550 BioLegend 367148 63D3 2.5 ul 3.3 ul
19 Anti-CD21-FITC BioLegend 354910 Bu32 2 ul 1.6 ul
20 Live dead dye- Blue BioLegend L23105 1:800 in PBS
21 CoV2-RBD-Biotin BioLegend 793906 2.5 ul of conjugated antibody 2.5 ul of conjugated antibody
22 SAv-BV421 BioLegend 405225 conjugated to CoV2-RBD-Biotin
23 CoV2-S1-Biotin BioLegend 793806 2.5 ul of conjugated antibody 2.5 ul of conjugated antibody
24 Anti-CD38-APC-Fire810 BioLgend 303550 HIT2 1ul 1ul
25 Anti-IgA-VioBlue Miltenyi 130-113-479 IS11-8E10 2 ul 1.3 ul
26 SAv-APC ThermoFisher S32362 conjugated to CoV2-S1-Biotin
27 Anti-CD85j-Super bright 436 ThermoFisher 62-5129-42 HP-F1 12.5 ul 6.5 ul
28 Anti-FCRL4-PerCP-eFluor710 ThermoFisher 46-3079-42 413D12 5 ul 2.4 ul
29 Anti-Tbet-PE-Cy7 Biolegend 644824 4B10 1ul 1ul
30 Anti-CD45-PerCP ThermoFisher MHCD4531 HI30 1.2ul 1.2ul

Antibodies used

4. S1+ /S1- B cell sorting panel
Number Reagent Source Catalog number Clone Dilution (PBMC, 30mill in 200ul) Dilution (Adenoid & Tonsil, 30mill in 200ul)
1 Anti-CD19-PeCy7 BioLegend 302216 HIB19 1: 20 1: 20
2 Anti-CD56-BV510 BioLegend 362533 5.1H11 1: 10 1: 10
3 Anti-CD14-BV510 BioLegend 367124 63D3 1: 10 1: 10
4 Anti-CD3-Alexa Fluor 488 BioLegend 317310 OKT3 7: 100 1: 20
5 Anti-CD8-PE BioLegend 303804 QA18A37 1: 20 1: 20
6 Anti-CD95-PE/Dazzle 594 BioLegend 305634 DX2 1: 40 1: 40
7 CoV2-S1-Biotin BioLegend 793806 1: 5 of conjugated antibody 1: 5 of conjugated antibody
8 Sav-BV421 BioLegend 405225
9 Anti-CD45-PerCP-Vio 700 Miltenyi 130-110-636 REA747 1: 20 1: 20
10 Anti-CD4-APC-eFLuor 780 ThermoFisher 47-0048-42 OKT4 1: 10 1: 10
11 Live dead dye-Aqua ThermoFisher L34957 7: 200 7: 200
12 SAv-APC ThermoFisher S32362

5. CITE-Seq antibody panel
Number Reagent Source Catalog number Barcode Clone Dilution (ul/30mill PBMC in 200ul volume) Dilution (ul/30mill Adenoid &
Tonsil in 200ul volume)
1 TotalSeq™-C0384 anti-human IgD Antibody BioLegend 348245 CAGTCTCCGTAGAGT IA6-2 28 ul
2 TotalSeq™-C0389 anti-human CD38 Antibody BioLegend 303543 TGTACCCGCTTGTGA HIT2 56 ul
3 TotalSeq™-C0154 anti-human CD27 Antibody BioLegend 302853 GCACTCCTGCATGTA O323 14 ul
4 TotalSeq™-C0181 anti-human CD21 Antibody BioLegend 354923 AACCTAGTAGTTCGG Bu32 7 ul
5 TotalSeq™-C0053 anti-human CD11c Antibody BioLegend 371521 TACGCCTATAACTTG S-HCL-3 7 ul
6 TotalSeq™-C0828 anti-human CD307d (FcRL4) Antibody BioLegend 340213 CGATTTGATCTGCCT 413D12 8 ul 28 ul
7 TotalSeq™-C0829 anti-human CD307e (FcRL5) Antibody BioLegend 340309 TCACGCAGTCCTCAA 509f6 4 ul 14 ul
8 TotalSeq™-C0826 anti-human CD307c/FcRL3 Antibody BioLegend 374413 GCCTAGTTTGAACGC H5/FcRL3 8 ul 28 ul
9 TotalSeq™-C0006 anti-human CD86 Antibody BioLegend 305447 GTCTTTGTCAGTGCA IT2.2 7 ul
10 TotalSeq™-C0147 anti-human CD62L Antibody BioLegend 304851 GTCCCTGCAACTTGA DREG-56 7 ul
11 TotalSeq™-C0146 anti-human CD69 Antibody BioLegend 310951 GTCTCTTGGCTTAAA FN50 56 ul
12 TotalSeq™-C0159 anti-human HLA-DR Antibody BioLegend 307663 AATAGCGAGCAAGTA L243 56 ul
13 TotalSeq™-C0161 anti-human CD11b Antibody BioLegend 301359 GACAAGTGATCTGCA ICRF44 Not added in 306-3 and 306-4
14 TotalSeq™-C0359 anti-human CD83 Antibody BioLegend 305341 CCACTCATTTCCGGT HB15e 28 ul
15 TotalSeq™-C0088 anti-human CD279 (PD-1) Antibody BioLegend 329963 ACAGCGCCGTATTTA EH12.2H7 28 ul
16 TotalSeq™-C0168 anti-human CD57 Recombinant Antibody BioLegend 393321 AACTCCCTATGGAGG "
QA17A04" 56 ul
17 TotalSeq™-C0145 anti-human CD103 (Integrin αE) Antibody BioLegend 350233 GACCTCATTGTGAAT Ber-ACT8 28 ul
18 TotalSeq™-C0143 anti-human CD196 (CCR6) Antibody BioLegend 353440 GATCCCTTTGTCACT G034E3 28 ul
19 TotalSeq™-C0140 anti-human CD183 (CXCR3) Antibody BioLegend 353747 GCGATGGTAGATTAT "
G025H7 " 56 ul
20 TotalSeq™-C0144 anti-human CD185 (CXCR5) Antibody BioLegend 356939 AATTCAACCGTCGCC J252D4 28 ul

Antibodies

Antibodies used

6. B cell sorting panel

Number Reagent Source Catalog number Clone (Tonsil, 75mill in total 200ul total staining volume including cells)
1Anti-CXCR3-PeCy5 BD5511281C625ul
2Anti-CD19-APCef780 ThermoFisher 47-0199-42 HIB19 25ul
3Live Dead Aqua ThermoFisher L34957 8ul
4Anti-CD21-FITCBioLegend 354910Bu32 7ul
5Anti-CD3-APC BD 561804 HIT3a 5ul
6Anti-CD38-BV421 BD562444HIT2 10ul
7Anti-IgD-BV605 BioLegend 348232IA6-210ul

7. BCR stimulation panel

Number Reagent Source
Catalog number Clone (Tonsil, ul/200ul)
1Anti-CD183(CXCR3)-PE-Cy5 BD5511281C6/CXCR35ul
2Anti-CXCR5-BV750 BD747111RF8B2 1ul
3Live dead dye-Blue BioLegend L231051:800 in PBS
4Anti-CD11c-BUV395 BD563787B-ly6 4.5ul
5Anti-CD69-BUV563 BD 748764FN501ul
6Anti-CD71-BUV661 BD749818L01.11.6ul
7Anti-CD21-FITC BioLegend 354910Bu32 1.6ul
8SAv-BV421 BioLegend 405225 conjugated to CoV2-RBD-Biotin
9Anti-CD3-BV570 BioLegend 300436UCHT1 3.3ul
10Anti-IgD-BV605 BioLegend 348232IA6-22.5ul
11Anti-CD19-BV650 BD 563226SJ25C1 7.5ul
12Anti-CD27-BV785 BioLegend 302832 O323 1.6ul
13Anti-CD14-Spark Blue 550 BioLegend 36714863D3 3.3ul
14Anti-CD45-PerCPThermoFisher MHCD4531 HI30 1.2ul
15Anti-CD95-BB700 BD566542DX2 1ul
16Anti-FCRL4-PerCP-eFluor710ThermoFisher 46-3079-42 413D12 2.4ul
17Anti-CD85j-Super bright 436 ThermoFisher 62-5129-42 HP-F1 6.5ul
18Anti-FCRL5/FCRL3-AF647 BD564343CD307e/c 4.5ul
19Anti-CD20-APC-H7 BD5607342H7 3.3ul
20Anti-CD38-APC-Fire810BioLgend 303550HIT2 1ul
21 phosphorylated PLCγ2 (p-Y759)-AF488 BD 558507 2ul
22phosphorylated Syk (p-Y348)-PE BD5585294ul
23CoV2-RBD-Biotin BioLegend 7939062.5 ul of conjugated antibody
24CoV2-S1-Biotin BioLegend 7938062.5 ul of conjugated antibody
25goat F(ab')2 anti-human IgA/G/MJackson ImmunoResearch Laboratories109-006-064 10ug/ml

Antibodies

Antibodies used

8. PB/PC differentiation panel
Number Reagent Source Catalog number Clone (Tonsil, ul/100ul)
1 Anti-CD21-BUV805 BUV805 742008 B-ly4 1ul
2 Anti-CD3-BV570 BioLegend 300436 UCHT1 2.5ul
3 Anti-IgD-BV605 BioLegend 348232 IA6-2 2ul
4 Anti-CD19-BV650 BD 563226 SJ25C1 3ul
5 Anti-CD27-BV785 BioLegend 302832 O323 2ul
6 Anti-CD20-APC-H7 BD560734 2H7 2ul
7 Anti-CD38-APC-Fire810 BioLgend 303550 HIT2 1ul
8 Anti-CD183(CXCR3)-PE-Cy5 BD 551128 1C6/CXCR3 5ul
9 Anti-IgG-PE-Cy7 BD 561298 G18-145 1ul
10 Anti-IgM-BV711 BioLegend 314540 MHM-88 2ul
11 Anti-IgA-VioBlue Miltenyi 130-113-479 IS11-8E10 1ul
12 R848 (Resiquimod) InvivoGen tlrl-r848-5 2.5ug/ml
13 CellTrace™ CFSE Cell Proliferation Kit ThermoFisher C34554 1:10000
14 Recombinant human IL-2 StemCell Technologies 78036.2 1000U/ml
15Live dead dye-Blue ThermoFisher L23105 1:800
16 Dynabeads™ CD19 Pan BThermo Scientific 11143D
17 FACS™ Lysing Solution 10X Concentrate BD 349202
18 FACS™ Permeabilizing Solution 2 BD 347692

9. Imaging panel
Number Reagent Source Catalog number Clone Concentration or dilution
1 Anti-BCL-6 Biocare Medical CM410-C LN22 1:15
2 Anti-CD11c Novus NBP3-14718 BLR138H 1:2000
3 Anti-CD138 R&D AF2780 Polyclonal Goat IgG 1:500
4 Anti-CD4-Alexa 700 R&D FAB8165N Polyclonal Goat IgG 1:20
5 Anti-CD20-eFluor 615 eBioscience 42-0202-82 L26 1:200
6 Anti-CXCR3-PE BD Bioscience 550633 1C6 1:50
7 Anti-IgD-Alexa 488a AbCam ab124795 EPR6146 1:20
8 Goat anti-mouse IgG, Alex 647 SouthernBiotech 1030-31 NA 1:400
9 Donkey anti-goat IgG (H+L), Alex 750 AbCam ab175745 NA 1:200
10 Donkey anti-rabbit IgG (H+L), Alex plus 405 ThermoFisher A48258 NA 1:500
11 TOPRO ThermoFisher T3602 NA 1:15000

Validation

All antibodies used for flow cytometry, CITE-seq, histo-cytometry and functional assays were from manufacturers/companies used in immunology studies. These companies have validated their antibodies in human PBMCs, tissues, or cell lines and information is available on their website.

All FACS antibodies were titrated for each panel and tissue to maximize specific signal and minimize background separately for PBMC or adenoid/tonsil cells. Control samples were undertaken for SARS-CoV-2 spike/RBD protein staining and Omi-RBD protein staining and are listed in Supplementary Data 11 (sheet 1) and shown in Figure 1e and Supplemental Figure 1b.

Antibody concentrations used for CITE-seq were optimized based on titration from flow cytometry. Isotype controls were included in CITE-seq and listed in Supplementary Data 11 (sheet 5) We have not independently verified the specificity of each antibody in our CITE-seq panel.

Eukaryotic cell lines

Policy information about cell lines and Sex and Gender in Research

Cell line source(s)

Authentication

Mycoplasma contamination

Commonly misidentified lines
(See ICLAC register)

Palaeontology and Archaeology

Specimen provenance

Specimen deposition

Dating methods

☐ Tick this box to confirm that the raw and calibrated dates are available in the paper or in Supplementary Information.

Ethics oversight

Note that full information on the approval of the study protocol must also be provided in the manuscript.

Animals and other research organisms

Policy information about studies involving animals; ARRIVE guidelines recommended for reporting animal research, and Sex and Gender in Research

Laboratory animals

Wild animals

Reporting on sex

Field-collected samples

Ethics oversight

Note that full information on the approval of the study protocol must also be provided in the manuscript.

Clinical data

Policy information about clinical studies
All manuscripts should comply with the ICMJE guidelines for publication of clinical research and a completed CONSORT checklist must be included with all submissions.

Clinical trial registration

Study protocol

Data collection

Outcomes

Dual use research of concern

Policy information about dual use research of concern

Hazards

Could the accidental, deliberate or reckless misuse of agents or technologies generated in the work, or the application of information presented in the manuscript, pose a threat to:

No	Yes	
☒	☐	Public health
☒	☐	National security
☒	☐	Crops and/or livestock
☒	☐	Ecosystems
☒	☐	Any other significant area

Experiments of concern

Does the work involve any of these experiments of concern:

No	Yes	
☒	☐	Demonstrate how to render a vaccine ineffective
☒	☐	Confer resistance to therapeutically useful antibiotics or antiviral agents
☒	☐	Enhance the virulence of a pathogen or render a nonpathogen virulent
☒	☐	Increase transmissibility of a pathogen
☒	☐	Alter the host range of a pathogen
☒	☐	Enable evasion of diagnostic/detection modalities
☒	☐	Enable the weaponization of a biological agent or toxin
☒	☐	Any other potentially harmful combination of experiments and agents

Plants

Seed stocks

Novel plant genotypes

Authentication

ChIP-seq

Data deposition

☐ Confirm that both raw and final processed data have been deposited in a public database such as GEO.

☐ Confirm that you have deposited or provided access to graph files (e.g. BED files) for the called peaks.

Data access links
May remain private before publication.

Files in database submission

Genome browser session
(e.g. UCSC)

Methodology

Replicates

Sequencing depth

Antibodies

Peak calling parameters

Data quality

Software

Flow Cytometry

Plots

Confirm that:

☒ The axis labels state the marker and fluorochrome used (e.g. CD4-FITC).

☒ The axis scales are clearly visible. Include numbers along axes only for bottom left plot of group (a 'group' is an analysis of identical markers).

☒ All plots are contour plots with outliers or pseudocolor plots.

☒ A numerical value for number of cells or percentage (with statistics) is provided.

Methodology

Sample preparation	Cell thawing was detailed in our recently published method paper (doi:10.3791/67188). The flow staining for each assay was provided in the methods.
Instrument	Aurora, Cytek
Software	Flowjo v10.9.0
Cell population abundance	Cells were not sorted before flow cytometry was undertaken.
Gating strategy	All cells were gated on a starting population of Live singlets. Individual population gating strategies are described in supplemental figures.

☒ Tick this box to confirm that a figure exemplifying the gating strategy is provided in the Supplementary Information.

Magnetic resonance imaging

Experimental design

Design type

Design specifications

Behavioral performance measures

Imaging type(s)

Field strength

Sequence & imaging parameters

Area of acquisition

Diffusion MRI ☐ Used ☐ Not used

Preprocessing

Preprocessing software

Normalization

Normalization template

Noise and artifact removal

Volume censoring

Statistical modeling & inference

Model type and settings

Effect(s) tested

Specify type of analysis: ☐ Whole brain ☐ ROI-based ☐ Both

Statistic type for inference

(See Eklund et al. 2016)

Correction

Models & analysis

n/a	Involved in the study
☐	☐ Functional and/or effective connectivity
☐	☐ Graph analysis
☐	☐ Multivariate modeling or predictive analysis

Functional and/or effective connectivity

Graph analysis

Multivariate modeling and predictive analysis

This checklist template is licensed under a Creative Commons Attribution 4.0 International License, which permits use, sharing, adaptation, distribution and reproduction in any medium or format, as long as you give appropriate credit to the original author(s) and the source, provide a link to the Creative Commons license, and indicate if changes were made. The images or other third party material in this article are included in the article's Creative Commons license, unless indicated otherwise in a credit line to the material. If material is not included in the article's Creative Commons license and your intended use is not permitted by statutory regulation or exceeds the permitted use, you will need to obtain permission directly from the copyright holder. To view a copy of this license, visit http://creativecommons.org/licenses/by/4.0/
