# Supplementary material for: SARS-CoV-2 infection and vaccination elicit distinct pharyngeal mucosal B cell responses in children
Source: Nat Commun. 2026 May 22;17:6924. doi: 10.1038/s41467-026-72996-3 (PMC13388998; doi:10.1038/s41467-026-72996-3)
Supplement: Supplementary file 6 — Transparent Peer Review file [file 41467_2026_72996_MOESM6_ESM.pdf]

nature portfolio

Peer Review File

SARS-CoV-2 infection and vaccination elicit distinct pharyngeal mucosal B cell responses in children

Corresponding Author: Dr Kalpana Manthiram

This manuscript has been previously reviewed at another journal. This document only contains information relating to versions considered at Nature Communications.

This file contains all reviewer reports in order by version, followed by all author rebuttals in order by version.

Version 2:

Reviewer comments:

Reviewer #1

(Remarks to the Author)
The authors addressed the concerns that I previously raised

(Remarks on code availability)

Reviewer #2

(Remarks to the Author)
The authors should be commended for their careful review of the comments and responsive alteration of the work. It is my view that the updated version of the work is suitable for publication in Nature Communications.

(Remarks on code availability)
This code does not seem to live at the indicated location.

Open Access This Peer Review File is licensed under a Creative Commons Attribution 4.0 International License, which permits use, sharing, adaptation, distribution and reproduction in any medium or format, as long as you give appropriate credit to the original author(s) and the source, provide a link to the Creative Commons license, and indicate if changes were made.
In cases where reviewers are anonymous, credit should be given to 'Anonymous Referee' and the source.
The images or other third party material in this Peer Review File are included in the article's Creative Commons license, unless indicated otherwise in a credit line to the material. If material is not included in the article's Creative Commons license and your intended use is not permitted by statutory regulation or exceeds the permitted use, you will need to obtain permission directly from the copyright holder.
To view a copy of this license, visit https://creativecommons.org/licenses/by/4.0/

Reviewer #1 (Remarks to the Author):

The authors addressed the concerns that I previously raised

Reviewer #2 (Remarks to the Author):

The authors should be commended for their careful review of the comments and responsive alteration of the work. It is my view that the updated version of the work is suitable for publication in Nature Communications.

Reviewer #2 (Remarks on code availability):

This code does not seem to live at the indicated location.

We appreciate the comments of the reviewers and their help to strengthen our manuscript. We have checked the github site with our code and have made it accessible to the public. The prior version may have had restricted access and so that may be why reviewer could not access it.

Please let us know if there are any other concerns.

Sincerely,

Kalpana Manthiram and Pamela Schwartzberg
